# Phylodynamics of foot-and-mouth disease virus O/PanAsia in Vietnam 2010–2014

**DOI:** 10.1186/s13567-017-0424-7

**Published:** 2017-04-13

**Authors:** Barbara Brito, Steven J. Pauszek, Michael Eschbaumer, Carolina Stenfeldt, Helena C. de Carvalho Ferreira, Le T. Vu, Nguyen T. Phuong, Bui H. Hoang, Nguyen D. Tho, Pham V. Dong, Phan Q. Minh, Ngo T. Long, Donald P. King, Nick J. Knowles, Do H. Dung, Luis L. Rodriguez, Jonathan Arzt

**Affiliations:** 1 Foreign Animal Disease Research Unit, Plum Island Animal Disease Center, ARS, USDA, Orient Point, NY USA; 2 Oak Ridge Institute for Science and Education, PIADC Research Participation Program, Oak Ridge, TN USA; 3 Regional Animal Health Office No. 6, Department of Animal Health, Ministry of Agriculture and Rural Development, Ho Chi Minh City, Vietnam; 4 National Centre for Veterinary Diagnostics, Hanoi, Vietnam; 5 Department of Animal Health, Ministry of Agriculture and Rural Development, Hanoi, Vietnam; 6 The Pirbright Institute, Pirbright, UK; 7 Friedrich-Loeffler-Institut, Federal Research Institute for Animal Health, Insel Riems, Germany

## Abstract

Foot-and-mouth disease virus (FMDV) is endemic in Vietnam, a country that plays an important role in livestock trade within Southeast Asia. The large populations of FMDV-susceptible species in Vietnam are important components of food production and of the national livelihood. In this study, we investigated the phylogeny of FMDV O/PanAsia in Vietnam, reconstructing the virus’ ancestral host species (pig, cattle or buffalo), clinical stage (subclinical carrier or clinically affected) and geographical location. Phylogenetic divergence time estimation and character state reconstruction analyses suggest that movement of viruses between species differ. While inferred transmissions from cattle to buffalo and pigs and from pigs to cattle are well supported, transmission from buffalo to other species, and from pigs to buffalo may be less frequent. Geographical movements of FMDV O/PanAsia virus appears to occur in all directions within the country, with the South Central Coast and the Northeast regions playing a more important role in FMDV O/PanAsia spread. Genetic selection of variants with changes at specific sites within FMDV VP1 coding region was different depending on host groups analyzed. The overall ratio of non-synonymous to synonymous nucleotide changes was greater in pigs compared to cattle and buffalo, whereas a higher number of individual amino acid sites under positive selection were detected in persistently infected, subclinical animals compared to viruses collected from clinically diseased animals. These results provide novel insights to understand FMDV evolution and its association with viral spread within endemic countries. These findings may support animal health organizations in their endeavor to design animal disease control strategies in response to outbreaks.

**Electronic supplementary material:**

The online version of this article (doi:10.1186/s13567-017-0424-7) contains supplementary material, which is available to authorized users.

## Introduction

Foot-and-mouth disease (FMD) is a highly transmissible viral disease of cloven hooved animals and is considered one of the most important diseases of livestock. Countries in Southeast and East Asia have varying levels of FMD endemicity, with Cambodia, Thailand, Laos, China and Vietnam having relatively high FMD incidence throughout the year [1]. Vietnam is the largest pig trader in Southeast Asia, so FMD control in this country is critical for the entire region [2]. The economic burden of FMD is substantial for large and small-scale pig producers and pig owners [3]. Cattle and water buffalo (*Bubalus bubalis*) have similar and important roles in agricultural practice in Vietnam [2, 4]. Both species are kept in varying degrees of intensity for dairy and meat production and are additionally used for draught purposes. Both species are often allowed to range freely for variable periods of time, and are frequently moved across country borders [5].

A study carried out in Vietnam, targeting areas with recent history of FMDV, found 22.3% seropositivity (to non-structural proteins) amongst subclinical buffalo and cattle sampled [6]. FMDV serotypes A and O currently circulate in Vietnam, while serotype Asia 1 has not been reported in Vietnam since 2008 [7–9]. FMDV serotype O is the most prevalent in the country; a recent study sequenced 71 serotype O viruses from samples collected between 2009 and 2013 in Vietnam, 65 of these viruses belonged to O/ME-SA/PanAsia lineage, while only 6 were classified as O/SEA/Mya-98 [6, 8]. However, in Vietnam from 2014–2016 it is believed that there has been a resurgence of O/SEA/Mya-98 (Dung and Long, unpublished data). Additionally, an incursion of O/ME-SA/Ind2001d lineage was reported for the first time in the country in 2016 [10].

Ruminants infected with FMDV may either clear the virus within 1–2 weeks after initial infection or develop a subclinical, persistent infection [11–13]. The World Organization for Animal Health (OIE) defines persistent FMDV infection as the recovery of FMDV from oropharyngeal fluids >28 days post-infection (dpi). However, recent work has demonstrated that, under experimental conditions, cattle that clear the infection can be differentiated from those that develop persistent infection at 14 dpi for vaccinated animals and 21 dpi amongst non-vaccinated animals [14, 15]. These persistently infected animals are referred to as FMDV carriers [13, 16] and earlier studies have estimated that the proportion of infected cattle that become carriers range from 50 to 65% [14, 17]. The role of carriers in disease transmission amongst cattle has been extensively debated [17–19]. Researchers have conducted numerous experimental studies, but have failed to detect transmission from carrier cattle to susceptible animals, while others have concluded that transmission from persistently infected cattle may occur to a very limited extent [20, 21].

There are intrinsic challenges to investigating FMDV transmission between herds and between different host species under field conditions in areas where animal movements and husbandry practices are variable and inconsistently regulated. Molecular techniques used to study the evolution of a pathogen through phylogenetic reconstruction can help understand the geographical spread, transmission between species and transmission from carrier and acutely infected animals, respectively when records about animal movements and contacts are not available [22, 23].

The objective of this study was to reconstruct the phylogeny, inferred inter-species transmission, and geographic spread of FMDV using VP1 coding sequences obtained from different host species, clinical stages, and locations in Vietnam. These analyses can help identify characteristics of the host species, geographic location, and disease status that are associated with specific changes in the viral genome and selection pressures.

## Materials and methods

### Data source

This investigation of FMDV phylodynamics in Vietnam was based on 125 FMDV VP1 coding region sequences [639 nucleotides (nt) total length]. The VP1 capsid protein comprises approximately 7.6% of the FMDV genome, and is commonly used for first-line phylogenetic analyses because it is known to be the most variable region of FMDV due to selective pressure on the immunogenic epitopes contained therein [24]. These sequences were obtained from three sources: (1) previous studies conducted by our group wherein 77 FMDVs collected in Vietnam were sequenced at the Plum Island Animal Disease Center, United States and identified as FMDV O/ME-SA topotype, PanAsia lineage as described in previous publications [6, 25], (2) eleven sequences generated by the OIE/FAO World Reference Laboratory for Foot-and-mouth disease (WRLFMD, The Pirbright Institute, UK), delivered directly to the Vietnam Department of Animal Health and deposited in GenBank for this study, and (3) 37 recently described, genetically related sequences that were retrieved from GenBank, 34 of which are from Vietnam, two from Kazakhstan and one from China. All sequences used in the study are currently available (see Additional file 1; Table 1). No animal experimentation or euthanasia was performed for the sake of completing this study. Because several VP1 sequences were shorter than complete length of the protein-coding segment (639 nt), we trimmed the alignment to the first 621 nt, to have consistent data for phylogenetic reconstruction.

**Table 1:** **Bayesian stochastic search variable selection analysis results**

Discrete character	Bayes factor
Species	
Cattle to pig	5900
Cattle to buffalo	5900
Pig to cattle	1179
Persistent and outbreak	
Carrier to clinical	871
Clinical to carrier	871

Significant (Bayes factor > 3) non-zero transmission rates between species and between outbreak and persistent animals are shown.

### Phylogenetic reconstruction using divergence time estimation

We reconstructed the phylogeny of FMDV and estimated divergence times. Sequences were aligned using the MUSCLE algorithm [26]. To estimate the best codon partition and best substitution model, we analyzed the sequences using Partition Finder [27] and selected the best partition scheme based on the Bayesian Information Criterion (BIC). The purpose of this selection is to identify the best codon partition scheme and the best nt substitution model for each partition.

Using the best partition scheme and corresponding substitution model, we reconstructed the phylogeny of O/ME-SA/PanAsia FMD virus using a Bayesian statistical approach (Bayesian Evolutionary Analysis by Sampling Trees), implemented in BEAST 1.8.2 [28]. The sampling dates were specified to estimate times of divergence. We used the lognormal uncorrelated relaxed clock model, and the Coalescent Bayesian Skyline tree prior. The analysis was run for 2 × 10^8^ iterations within a web-based platform with access to computational resources available in CIPRES [29]. Convergence of the chain was assessed using Tracer 1.6, by visualizing traces of parameters of trees sampled and confirming that mixing of the chain had been achieved so that the effective sample size of all parameters was >200 [30]. From all trees sampled, the maximum clade credibility tree (MCC) was annotated and depicted using FigTree 1.4.2 [31]. The initial 10% sampled trees were discarded as burn-in. Time to most recent common ancestor (tMRCA) of all nodes and 95% highest posterior densities (95% HPD) were obtained from the MCC tree.

### Phylogeographic analysis and ancestral character state reconstruction

We used discrete ancestral character state reconstruction to estimate the viral history, specifying traits according to 3 different characteristics: the host species, the clinical status, and the location. To reconstruct the host species, we defined three discrete characters: cattle, pig or buffalo. For the clinical status, we used two characters: sequences described as “Clinical” were obtained from samples derived from vesicular lesions of animals during outbreaks of clinical FMD in Vietnam, whereas “Carrier” sequences were obtained from oropharyngeal fluid (probang) from subclinical cattle and buffalo identified through active surveillance as previously described [6]. To infer ancestral states with respect to geographical location we categorized each viral sequence assigning one of the 8 different geographical regions defined within Vietnam: Northeast, Northwest, Red River Delta, North Central Coast, South Central Coast, Central Highlands, Southeast, and the Mekong River Delta.

For each host species, clinical disease and geographic location traits, we reconstructed the inferred character state for each node within the phylogenetic tree. We analyzed the inferred transmission rates between character states using an asymmetric model for the discrete traits and estimated the significance of the network with Bayesian stochastic search variable selection (BSSVS), which tests the hypothesis of non-zero transmission rates between discrete characters [22]. Statistical support was assessed using Bayes Factor (BF) for discrete traits implemented in SPREAD1.0.6 [32], we considered BF > 3 as significant non-zero transmission. The analysis was carried out in BEAST 1.8.2 and the number of iterations and assessment of chains were performed as described above. Additionally, we obtained the count of character transitions (“jumps”) from all trees (excluding initial burning). The 95% high-density interval (95% HDI) of the values collected for each character state change that had a non-zero rate BF > 3.0 was computed using HDInterval package in R [33].

### Evolutionary selection of nucleotide sites across different species and between clinically versus persistently infected animals

The VP1 coding region of the 122 FMDV O/ME-SA/PanAsia sequences described above and the additional related sequences (*n* = 3) from Kazakhstan and China were analyzed for positive and negative selection. Sequence groups from different species (cattle, pigs and buffalo-excluding samples from persistently infected animals) as well as clinical or persistent status were analyzed independently to study differences in viral selection by nt site and overall selection. The mean ratio of non-synonymous (dN) and synonymous changes (dS) (global ω = dN/dS ratio) was computed using the single-likelihood ancestor counting (SLAC) method, and 95% confidence intervals estimated from the data and the likelihood profile [34]. Individual site selection was also computed by the fixed effects likelihood (FEL) method and by the random effects likelihood (REL; which was used for the species groups but not for the clinical/carrier groups due to the size restriction -number of sequences- of the analysis) [34]. Only unique sequences were used in this analysis (i.e. identical sequences were removed). The HKY85 nt substitution model was used. A site was considered positively or negatively selected if identified by at least one of the methods described (dN-dS > 0 with a *p* value cutoff 0.1 for SLAC, 0.1 for FEL and 50 BF for REL). We displayed the dN-dS values computed for each codon position and indicated the statistically significant positively selected sites. The analyses were performed in the HyPhy2.2.1 software package [34, 35].

## Results

### Divergence time estimation and ancestral character reconstruction

The partition scheme and substitution models selected were the K80 + I for codon positions 1 + 2, and the HKY + G substitution model, for codon position 3. The mean substitution/site/year for VP1 coding segment the O/ME-SA/PanAsia phylogeny reconstructed was 1.66 × 10^−2^ (95% HPD 1.21–2.11 × 10^−2^).

### Ancestral state character reconstruction

#### Host species

**Figure 1:**
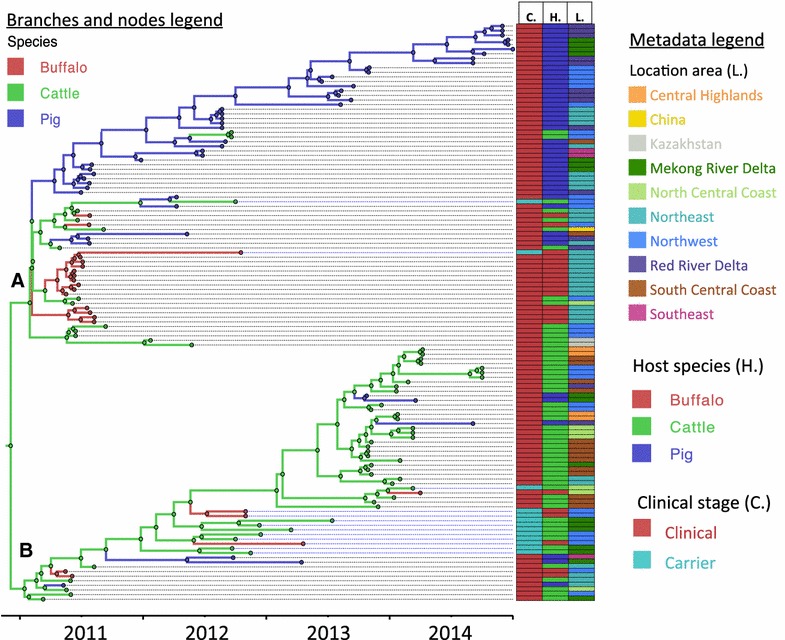
**Maximum clade credibility tree of FMDVs in Vietnam and related viruses from China and Kazakhstan between 2010 and 2014.** The color of tree branches and nodes indicates the ancestral host species for the reconstructed phylogeny. Clades **A** and **B** represent the two main O/ME-SA/PanAsia sublineages that have diverged recently in Vietnam. Characteristics of the sampled viruses (clinical stage: C., host species: H., and location: L.) are indicated in colored columns aligned to the right of the tree, color coding is indicated in the metadata legend.

The MCC tree depicting the reconstruction based on FMDV host species is presented in Figure 1. The current FMD O/ME-SA/PanAsia viruses circulating in Vietnam diverged into two different clades in June 2010 (95% HPD March 2010 to September 2010). One of these lineages (Figure 1, branch A), initially found in all three hosts species, diverged into a cluster of viruses that subsequently were found predominantly in pigs. Transitions between host species character state inferred from the MCC tree (and 95% HDI) occurred from cattle to pigs (7 events 95% HDI 5–10), pigs to cattle (2 events 95% HDI 1–3), and from cattle to buffalo (8 events 95% HDI 7–11). Results of the analysis to estimate significant transmission rates between species, BSSVS, are shown in Table 1. With respect to host species, non-zero inferred transmission rates were detected from cattle to pig, from cattle to buffalo and from pig to cattle.

#### Clinical status

**Figure 2:**
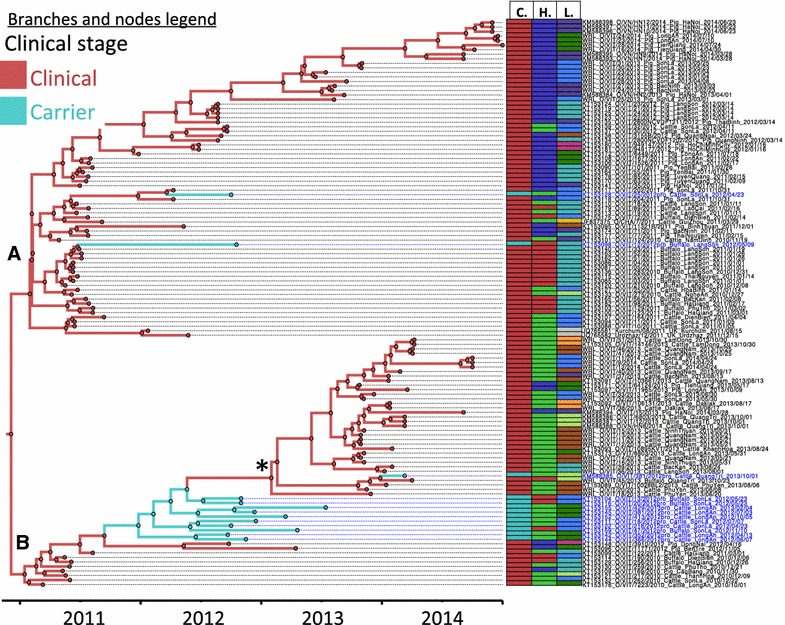
**Maximum clade credibility FMDV O/ME-SA/PanAsia viruses collected in Vietnam (and additional 3 sequences from China and Kazakhstan) between 2010 and 2014.** Nodes and branches of the trees are colored according to the clinical stage reconstructed in the phylogeny. The ancestral reconstruction of the viruses analyzed suggests 1 instance where outbreak viruses may have originated from carriers (*). Clades **A** and **B** represent the two main O/ME-SA/PanAsia sublineages that have diverged recently in Vietnam. Characteristics of the sampled viruses (clinical stage: C., host species: H., and location: L.) are indicated in colored columns aligned to the right of the tree (color coding legend for the columns is indicated in Figure 1).

The MCC tree showing the ancestral reconstruction of clinically affected animals (“Clinical”) and subclinically infected animals (“Carriers”) is shown in Figure 2. In the upper main branch of the tree (Figure 2, branch A), there are two viruses from persistently infected animals (KT153098-O/VIT/12/2012pro and KT153128-O/VIT/25/2012pro) likely originating from previous outbreaks. However the tMRCA with the most closely genetically related ‘clinical’ viruses was relatively long (compared to tMRCA of related viruses), suggesting that they had already diverged approximately one year (1.32 and 0.53 years for viruses O/VIT/12/2012pro and O/VIT/25/2012pro respectively) from their ancestral “clinical” sample sequences. The lower branch of the tree (Figure 2, branch B) includes closely related viruses from 9 different persistently infected animals. The ancestral reconstruction of the set of viruses analyzed suggests that these viruses may have initiated an outbreak with clinical disease, which includes several closely related viruses (Figure 2, *).

Inferred non-zero transmission rates estimated by BSSVS analysis (Table 1), which is used to detect significant non-zero transmission from carrier to clinical and from clinical to carrier categories.

### Phylogeographic analysis

**Figure 3:**
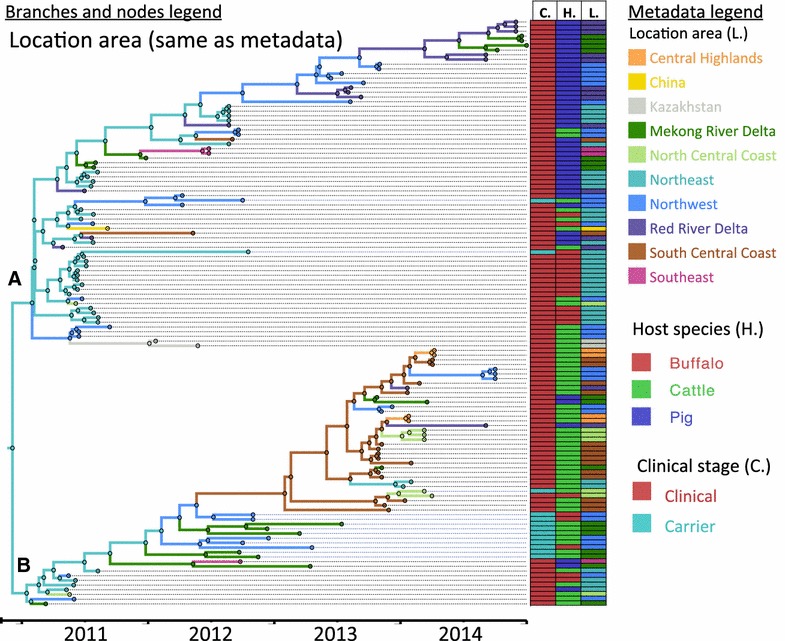
**Maximum clade credibility FMDV O/ME-SA/PanAsia viruses collected in Vietnam (and additional 3 sequences from China and Kazakhstan) between 2010 and 2014.** Nodes and branches of the trees are colored according to the location with the higher posterior probability in the reconstructed phylogeny. Ancestors of the two divergent clusters are inferred to exist in the Northeast. Clades **A** and **B** represent the two main O/ME-SA/PanAsia sublineages that have diverged recently in Vietnam. Characteristics of the sampled viruses (clinical stage: C., host species: H., and location: L.) are indicated in colored columns aligned to the right of the tree, color coding is indicated in the metadata legend.

The phylogeographic reconstruction of the viruses is shown in Figure 3. The common ancestor of all O/ME-SA/PanAsia sequences included in this study was located in the northeast region. Putative movements of this lineage from the Northeast of Vietnam into China and from the Northwest Region of Vietnam into Kazakhstan can be observed in Figure 3—branch A and may have occurred in December of 2010. The phylogeographic analysis demonstrates that the upper part of Figure 3—branch A, which contains a cluster of pig-derived viruses (Figure 2, branch A), may have initially originated from the Northeast and then spread into the Northwest, and the Red River Delta Regions.

**Figure 4:**
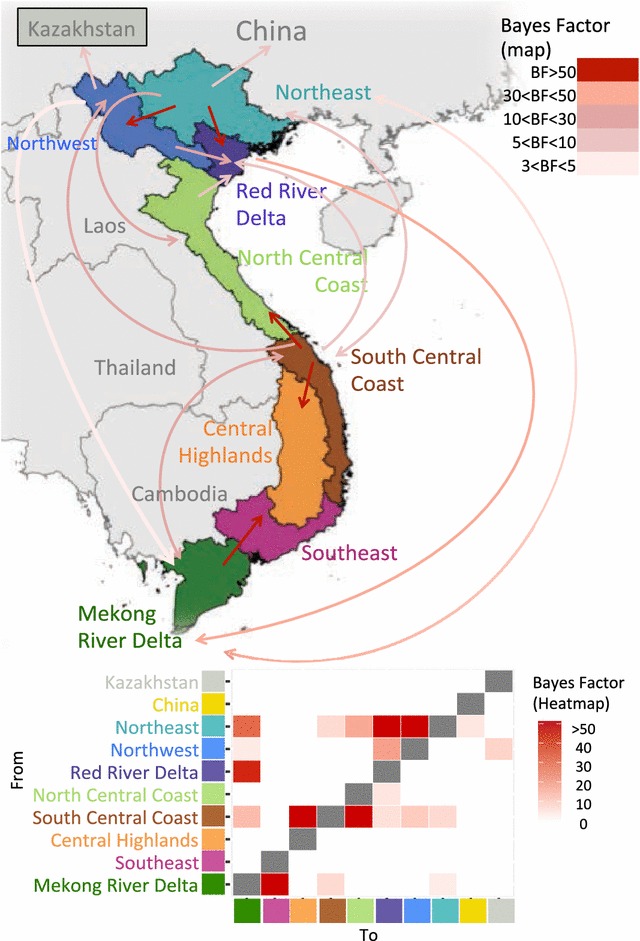
**Results from the Bayesian stochastic search variable selection of the Phylogeographic reconstruction of O/ME-SA/PanAsia in Vietnam regions, and related sequences from Kazakhstan and China.** Only Bayes factor > 3 are represented as arrows as significant non-zero transmission of O/ME-SA/PanAsia. Most significant transmissions were inferred for some adjacent regions, although inferred transmission between some distant regions was also statistically supported. The heatmap in the lower area of the figure depicts the magnitude of the statistical support (Bayes factor) for transmission rate between geographic regions in Vietnam. This heatmap allows visualizing that more transmission occurred from South Central Coast and the Northeast regions into other areas, whereas the Red River Delta and Mekong Delta were the ones with more incoming transmission from other regions.

To visualize the significant inferred transmission rates (BF > 3) between the geographical regions we overlaid these results on a map of Vietnam (Figure 4). The most significant inferred transmissions were detected from Northeast and South Central Coast to their corresponding adjacent regions, as well as transmission from Mekong River Delta into Southeast region. Inferred disease spread occurred in northern and southern directions, however there was a general trend of higher BF for southbound transfer of viruses. BF-inferred transmission is also shown in the heatmap displayed in Figure 4, and it further evidences that the South Central Coast and the Northeast are the regions from where the viruses are more frequently spread into other regions.

### Evolutionary selection of sites in different species and clinically versus persistently infected animals

Results of the global dN/dS ratio (ratio of non-synonymous to synonymous changes) estimated by category are shown in Table 2. Considering only viruses from clinical outbreaks, pig-derived viruses had a higher overall positive selection ratio (dN/dS) compared to buffalo and cattle; however, only the difference with cattle was statistically significant at *p* < 0.05 (based on non-overlapping 95% confidence intervals). The extent of positive selection was similar in buffalo and cattle. Similarly, viruses collected from persistently infected animals and from clinically affected ones had almost the same dN/dS values (Table 2).

**Table 2:** **Results of the global dN/dS ratio estimated for each of the categories and corresponding 95% confidence interval**

Category	ω = dN/dS ratio	Lower 95% CI	Upper 95% CI
Cattle^a^	0.161	0.107	0.23
Pig^a^	0.272	0.205	0.351
Buffalo^a^	0.181	0.094	0.312
Carrier^b^	0.160	0.112	0.222
Outbreak^b^	0.160	0.11	0.224
All	0.209	0.173	0.249

^a^Sequence from virus collected from clinical samples.

^b^Sequences from viruses collected from cattle and buffalo only.

**Figure 5:**
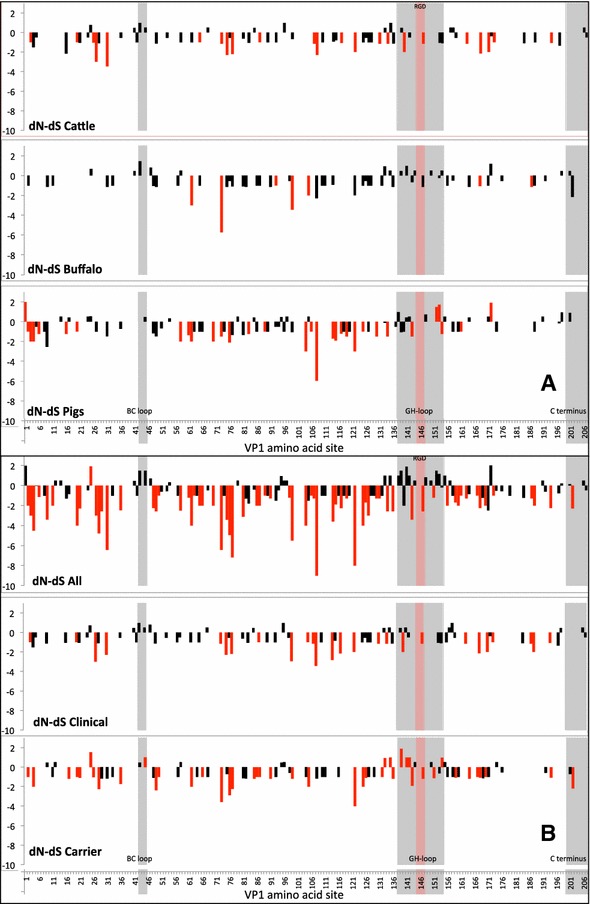
**Site selection (dN-dS) by VP1 coding region in alignments of FMDV sequences. A** Site selection (dN-dS) results per VP1 coding region in alignments of cattle viral sequences (excluding persistently infected), buffalo (excluding persistently infected) and pigs. Values >0 represent positive selection. Bars colored in red indicate sites where selection is statistically significant. Grey shaded areas correspond to known antigenic sites within VP1: BC loop (sites 43–45), GH loop (sites 238–254), and C terminus (sites 200–207). Pink-shaded sites correspond to the RGD integrin-binding motif. **B** Site selection (dN-dS) results per VP1 coding region in alignments including of all viral sequences, “clinical” (cattle and buffalo) viral sequences and “carrier” (cattle and buffalo) infected animals.

Results of the specific site selection for every codon (dN-dS) in all categories are shown in Figures 5A and B. No specific sites under positive selection were found to be statistically significant when analyzing viral sequences collected from cattle and buffalo, whereas few sites were detected when analyzing viral sequences from pig (site numbers: 1, 152, 153 and 172). Several statistically significant negatively selected sites were found for cattle (23 sites), pigs (28 sites), and buffalo (7 sites). Overall positive selection was mainly found within the known antigenic sites (GH loop, BC loop). Two statistically significant positively selected sites were found in the GH loop in pigs, compared to none in buffalo and cattle groups.

Although the global positive selection ratio was the same in carrier and clinically diseased animals, when dissecting the specific positively selected amino acid sites in the antigenic domains, carrier animals had a higher number of statistically significant positively selected sites compared to selection in viruses from clinical samples: Four statistically significant positively selected sites in the GH loop (near the RGD motif) and one in the BC loop were found in carriers, whereas two positively sites in the GH loop and two sites in the BC loop without statistical significance were found in clinical samples from cattle and buffalo (Figure 5B). Three additional sites of significant positive selection were also identified in viruses from carriers, but in non-antigenic regions. When analyzing all viral sequences together, several positively selected (non significant) sites were found within the GH-loop and the BC loop, whereas numerous significantly negatively selected sites were found throughout VP1. Site 73 was consistently negatively selected (higher expected number of synonymous changes) in all group categories. Only one site (154) was detected as both positive and negatively selected depending on the category; while positive selection was detected within the carrier animals category, this site was identified as a negatively selected for pig viral sequences.

## Discussion

In the current study, we analyzed phylodynamics of FMDV O/ME-SA/PanAsia viruses recovered from livestock in Vietnam between 2010 and 2014. The results presented herein provide a novel overview of comparative viral evolution of field samples collected from different host species, locations, and different clinical stages of infection. These data suggest differences in viral evolution within distinct animal groups, which may contribute to understanding of the mechanisms of maintenance, emergence and spread of viruses across Vietnam.

Using viruses sampled between 2010 and 2014 from different species, stages of infection and provinces of Vietnam, we found two main sublineages of FMDV O/ME-SA/PanAsia. One of these sublineages has been most frequently recovered from pigs since 2011 while the other main sublineage has been most frequently found in cattle. Some FMDV strains may have a predilection to certain host species; this phenomenon has been extensively described with porcinophilic FMDV serotype O of the Cathay topotype [36, 37]. However, the data presented herein is not sufficient to determine limited host range of the mostly pig-derived FMDV subclade found in this study. This is because several other factors, including sampling bias, could have determined the apparent species-specificity. The hypothesis of limited host range could be confirmed by follow up studies in tissue culture or in vivo to determine if these viruses have evolved to a specific pig host predilection.

The substitution rate calculated herein for VP1 of FMDV O/ME-SA/PanAsia circulating in Vietnam between 2009 and 2014 was similar to a previous estimate of serotype O Cathay topotype (1.06 × 10^−2^) [38]. However, the rate estimated for this study was higher than those computed across other serotype O viruses estimated at 6.65 × 10^−3^ [39], 6.34 × 10^−3^ [40], or 4.81 × 10^−3^ (serotype O collected globally between 1939 and 2010) [41] substitutions/site/year. The high mutation rate found in our analyses may be a result of capturing mutations that do not become fixed in a population, as a consequence of sampling a high number of outbreaks in a relatively short period of time. In contrast, isolated sequences from different regions sampled over longer periods may reflect fixed mutations, which may result in computing a different substitution rate. However, the specific underlying mechanisms driving these differences across studies using different geotemporal conditions remain undetermined.

Inferred transmission as deduced by ancestral character reconstruction suggests that cattle have a relevant role in inter-species dissemination. Various factors may contribute to this phenomenon including subclinical infection in vaccinated cattle and a longer duration of FMDV infectiousness in cattle compared to other species [12, 13]. Similarly, our study also provides evidence of inferred transmission of viruses from pigs to cattle. This may be explained by the large pig population in the country, and husbandry practices including the highly common practice of co-mingling pigs with other species in rural households and villages. In contrast, based on the sequences obtained, we did not identify putative transmission from buffalo to either cattle or pigs. Although transmission from cattle to buffalo, and buffalo to cattle is known to occur [42, 43], to our knowledge, comparative quantitative estimates of transmission rates of these two species have not been estimated. We detected several instances of transfer of viruses from cattle to buffalo, but none from buffalo to cattle. The lack of detection may either represent lesser ability of buffalo to transmit the disease to cattle, differential ranging and transport of buffalo and cattle within and between different areas in Vietnam, or it may be an effect of sampling bias.

Phylogenetic analyses in this study, suggest that there are instances in which viruses found in carrier animals may have been the ancestors of viruses that later caused outbreaks. However, it is also possible that undersampling of outbreak sequences around the period of the apparent carrier-to-clinical transfer may have created sampling bias which influenced these results (Figure 2). Transmission from carriers to susceptible animals has been either low or inexistent in controlled experiments. However, the potential epidemiological role of the FMDV carrier state in maintaining FMDV and being the source of new outbreaks is still highly controversial [13, 17, 44]. Quantitatively, it has been estimated that the transmission rate from acutely (clinically) infected animals is more than 500 times that of the carrier [20]. However even though the probability is exceedingly low, it would only require one successful transmission event within the thousands of contacts to start an outbreak and thus have a substantial impact.

Overall, the number of outbreaks and potential infected animals included herein is low compared to the actual number of outbreaks and infections that occurred in the field. Thus, such findings must be interpreted conservatively. Because of the inability to obtain every relevant virus from the field, analyses derived from studies such as this, contain intrinsic sampling bias and must be interpreted as generating, rather than confirming hypotheses. Similarly, these phylogenetic analyses were carried out using only VP1 coding sequence of FMDV, which is 639 nt long of the ~7000 nt ORF length. This genetic segment is the most variable within FMDV genome and contains the most relevant antigenic sites. Full genome sequences in sufficient quantities to enable these studies of phylodynamics of Vietnam, were not available at the time of this work.

Geographic spread of FMD within Vietnam inferred by viral sequence suggests that transmission occurs between several different regions of the country. However transmission seems to be more frequent from South Central Coast and Northeast into other parts of the country. As expected, the greatest extent of transmission was to immediately adjacent regions, but also to distant parts of the country. These findings are partially consistent with previous studies conducted by the OIE, which assessed the livestock movement in Vietnam and neighboring countries [5]. That report describes that substantial ruminant movement occurs from central areas of the country into northern and southern regions. Within the northern regions, this flow tends to be towards northeastern region, whereas in the south, animal movement converges into northern areas of the Mekong River Delta region. In contrast, pig movement occurs mostly from northern into southern areas of Vietnam and from northern areas into China. Transmission occurring in different geographic directions inferred by our analysis reflects the combination of these reported pig and ruminant species movement. The movement of large ruminants and pigs into China, however, is supported by our current inferred viral transmission data. Additionally, animal movement from Thailand, Cambodia and Laos into Vietnam occurs frequently [5].

Selection pressure may be an important driver of viral evolution [45]. It is biologically plausible that selection pressures differ in different host species, or through different phases of infection within the same host (e.g. acute clinical infection in contrast to persistent infection). Viral evolution comparing different groups of infected animals has not been extensively explored previously. Potential specific molecular changes in the FMDV genome that establish persistent infection have been reported in few controlled studies [17, 21, 46]. Here, we found that the global positive selection of VP1 tended to be similar in carrier animals compared to viruses recovered from clinical outbreaks. Although the value of the global positive selection rate was almost equal in carrier and outbreak groups, the quantity of individual (statistically significant) positive selected codon sites was higher in carrier animals compared with those with acute clinical infection, especially in the antigenic regions.

Because animal movement and mixing animals from different origins is a common practice in Vietnam, persistently infected animals may play an important role in disease spread. Understanding specific molecular (genomic and/or antigenic) changes can eventually help to understand the mechanisms through which FMDV persistence is established and maintained. Although analyses of our data demonstrate potentially relevant trends, results should be interpreted carefully with mindfulness of the relatively low quantity of samples from carrier animals (*n* = 12).

Additionally, we found that statistically significant positive selection was greatest in pigs compared to buffalo and cattle, and that these sites were found mostly in regions that code for the known antigenic domains. This suggests that the viral evolution in the pig population may provide an important contribution to antigenic diversity and strain emergence compared to other species. Previous work has shown that most of the genomic variation occurs early in the course of infection, when there is greater viral replication [47].

In conclusion, the current study suggests that inferred virus transmission patterns in Vietnam may differ depending on the host species and clinical status of infected hosts. This may be related to differences in viral selection between species and in the persistently infected animals compared to clinically affected individuals. These differences can help to elucidate viral evolution within-host, across host species, and within populations. Application and combination of the methodologies described herein to study specific aspects of FMDV evolution may help to gain new knowledge in our understanding of FMDV, ultimately contributing to disease control and eradication in endemic countries.

## Additional file

**Additional file 1.** **Foot-and-mouth disease viruses from Vietnam, Kazakhstan and China included in the study analyses.** Genbank accession numbers, virus name, geographical location, collection date, host species and clinical stage (outbreak or persistently infected animals) are tabulated.

## Abbreviations

BF: Bayes factor
BSSVS: Bayesian stochastic search variable selection
dN: substitution rates at non-synonymous sites
dpi: days post-infection
dS: substitution rates at synonymous sites
FMD: foot-and-mouth disease
FMDV: foot-and-mouth disease virus
MCC: maximum clade credibility
Nt: nucleotide
OIE: World Organization for Animal Health
tMRCA: time to most recent common ancestor
