# Supplementary material for: Phylodynamics of foot-and-mouth disease virus O/PanAsia in Vietnam 2010–2014
Source: Vet Res. 2017 Apr 13;48:24. doi: 10.1186/s13567-017-0424-7 (PMC5390394; doi:10.1186/s13567-017-0424-7)
Supplement: Supplementary file 1 — Additional file 1. Foot-and-mouth disease viruses from Vietnam, Kazakhstan and China included in the study analyses. Genbank accession numbers, virus name, geographical location, collection date, host species and clinical stage (outbreak or persistently infected animals) are tabulated. [file 13567_2017_424_MOESM1_ESM.docx]

**Additional file 1 Foot-and-mouth disease viruses from Vietnam, Kazakhstan and China included in the study analyses.**

| Virus name | Country | Adm. Area | Country Region | Host species | Collection Date | Clinical stage | Accsn |
| --- | --- | --- | --- | --- | --- | --- | --- |
| O/CHA/7/2011 | China | GuiZhou | China | Cattle | 29-Mar-11 | NA** | JF837375 |
| Kurchum/08/2011 | Kazakhstan | Kurchum | Kazakhstan | NA* | 15-Aug-11 | NA** | JQ765581 |
| Urdzhaz/12/2011 | Kazakhstan | Urdzhaz | Kazakhstan | NA* | 15-Dec-11 | NA** | JQ765582 |
| O/VN/HN2/2013 | Vietnam | HaNoi | Red River Delta | Pig | 1-Apr-13 | outbreak | KM588384 |
| O/VIT/381/2012pro | Vietnam | QuangTri | North Central Coastal | Cattle | 1-Oct-13 | NA** | KM588385 |
| O/VIT/6803/2013 | Vietnam | QuangTri | North Central Coastal | Cattle | 1-Oct-13 | NA** | KM588386 |
| O/VIT/12/2013 | Vietnam | QuangTri | North Central Coastal | Cattle | 1-Oct-13 | NA** | KM588387 |
| O/VN/QT9/2013 | Vietnam | QuangTri | North Central Coastal | Cattle | 1-Oct-13 | NA** | KM588388 |
| O/VN/HN7/2014 | Vietnam | HaNoi | Red River Delta | Pig | 28-Mar-14 | outbreak | KM588393 |
| O/VN/HN8/2014 | Vietnam | HaNoi | Red River Delta | Pig | 28-Mar-14 | outbreak | KM588394 |
| O/VN/HN9/2014 | Vietnam | HaNoi | Red River Delta | Pig | 28-Mar-14 | outbreak | KM588395 |
| O/VN/HN10/2014 | Vietnam | HaNoi | Red River Delta | Pig | 23-Jun-14 | outbreak | KM588396 |
| O/VN/HN11/2014 | Vietnam | HaNoi | Red River Delta | Pig | 23-Jun-14 | outbreak | KM588397 |
| O/VN/HN12/2014 | Vietnam | HaNoi | Red River Delta | Pig | 23-Jun-14 | outbreak | KM588398 |
| O/VIT/03/2011 | Vietnam | LangSon | North Eastern | Buffalo | 1-Jan-11 | outbreak | KT153085 |
| O/VIT/04/2011 | Vietnam | LangSon | North Eastern | Buffalo | 1-Jan-11 | outbreak | KT153086 |
| O/VIT/05/2011 | Vietnam | LangSon | North Eastern | Buffalo | 1-Jan-11 | outbreak | KT153087 |
| O/VIT/10/2011 | Vietnam | SonLa | North Western | Cattle | 5-Jan-11 | outbreak | KT153088 |
| O/VIT/10028BL2/2013 | Vietnam | PhuYen | South Central Coastal | Cattle | 6-Aug-13 | outbreak | KT153089 |
| O/VIT/101/2011 | Vietnam | YenBai | North Eastern | Pig | 28-Feb-11 | outbreak | KT153090 |
| O/VIT/103661/2013 | Vietnam | QuangNam | South Central Coastal | Cattle | 13-Aug-13 | outbreak | KT153091 |
| O/VIT/106131/2013 | Vietnam | Daklak | Central Highlands | Cattle | 17-Aug-13 | outbreak | KT153092 |
| O/VIT/10895KV1/2013 | Vietnam | KhanhHoa | South Central Coastal | Cattle | 24-Aug-13 | outbreak | KT153093 |
| O/VIT/11/2011 | Vietnam | SonLa | North Western | Cattle | 5-Jan-11 | outbreak | KT153094 |
| O/VIT/11321B/2011 | Vietnam | BinhThuan | South Central Coastal | Pig | 1-Dec-11 | outbreak | KT153095 |
| O/VIT/11771/2012 | Vietnam | BenTre | Mekong River Delta | Pig | 5-Nov-12 | outbreak | KT153096 |
| O/VIT/11965/2013 | Vietnam | LongAn | Mekong River Delta | Pig | 9-Oct-13 | outbreak | KT153097 |
| O/VIT/12/2012pro | Vietnam | LangSon | North Eastern | Buffalo | 9-May-12 | carrier | KT153098 |
| O/VIT/122/2011 | Vietnam | HaGiang | North Eastern | Cattle | 1-Mar-11 | outbreak | KT153099 |
| O/VIT/123/2011 | Vietnam | HaGiang | North Eastern | Buffalo | 1-Mar-11 | outbreak | KT153100 |
| O/VIT/124/2010 | Vietnam | NamDinh | Red River Delta | Cattle | 19-Nov-10 | outbreak | KT153101 |
| O/VIT/129/2011 | Vietnam | PhuTho | North Eastern | Buffalo | 2-Mar-11 | outbreak | KT153103 |
| O/VIT/13/2012pro | Vietnam | SonLa | North Western | Buffalo | 23-May-12 | carrier | KT153104 |
| O/VIT/14146/2013 | Vietnam | LamDong | Central Highlands | Cattle | 30-Oct-13 | outbreak | KT153105 |
| O/VIT/1526/2011 | Vietnam | LongAn | Mekong River Delta | Pig | 17-Feb-11 | outbreak | KT153106 |
| O/VIT/164/2011 | Vietnam | DienBien | North Western | Cattle | 4-Apr-11 | outbreak | KT153107 |
| O/VIT/1677/2011 | Vietnam | LongAn | Mekong River Delta | Pig | 22-Feb-11 | outbreak | KT153108 |
| O/VIT/169/2010 | Vietnam | CaoBang | North Eastern | Pig | 30-Nov-10 | outbreak | KT153109 |
| O/VIT/18/2011 | Vietnam | LangSon | North Eastern | Cattle | 11-Jan-11 | outbreak | KT153110 |
| O/VIT/18/2012pro | Vietnam | SonLa | North Western | Cattle | 7-Jul-12 | carrier | KT153111 |
| O/VIT/19/2011 | Vietnam | LangSon | North Eastern | Cattle | 11-Jan-11 | outbreak | KT153113 |
| O/VIT/190/2010 | Vietnam | DienBien | North Western | Buffalo | 6-Dec-10 | outbreak | KT153114 |
| O/VIT/20/2011 | Vietnam | ThaiNguyen | North Eastern | Buffalo | 14-Jan-11 | outbreak | KT153115 |
| O/VIT/20/2012pro | Vietnam | SonLa | North Western | Buffalo | 23-May-12 | carrier | KT153116 |
| O/VIT/202/2011 | Vietnam | SonLa | North Western | Pig | 31-Oct-11 | outbreak | KT153117 |
| O/VIT/204/2011 | Vietnam | SonLa | North Western | Pig | 31-Oct-11 | outbreak | KT153118 |
| O/VIT/21/2012 | Vietnam | LangSon | North Eastern | Pig | 14-Mar-12 | outbreak | KT153119 |
| O/VIT/210/2010 | Vietnam | LangSon | North Eastern | Buffalo | 8-Dec-10 | outbreak | KT153120 |
| O/VIT/217/2010 | Vietnam | ThanhHoa | North Central Coastal | Cattle | 9-Dec-10 | outbreak | KT153121 |
| O/VIT/218/2012pro | Vietnam | SonLa | North Western | Cattle | 23-Apr-12 | carrier | KT153122 |
| O/VIT/22/2012 | Vietnam | LangSon | North Eastern | Pig | 14-Mar-12 | outbreak | KT153123 |
| O/VIT/23/2012 | Vietnam | LangSon | North Eastern | Pig | 14-Mar-12 | outbreak | KT153124 |
| O/VIT/24/2012 | Vietnam | LangSon | North Eastern | Pig | 14-Mar-12 | outbreak | KT153125 |
| O/VIT/25/2011 | Vietnam | HoaBinh | North Western | Cattle | 14-Jan-11 | outbreak | KT153127 |
| O/VIT/25/2012pro | Vietnam | SonLa | North Western | Cattle | 23-Apr-12 | carrier | KT153128 |
| O/VIT/256/2010 | Vietnam | HaGiang | North Eastern | Buffalo | 26-Dec-10 | outbreak | KT153129 |
| O/VIT/259/2010 | Vietnam | PhuTho | North Eastern | Cattle | 21-Dec-10 | outbreak | KT153130 |
| O/VIT/262/2010 | Vietnam | SonLa | North Western | Cattle | 22-Dec-10 | outbreak | KT153132 |
| O/VIT/275/2010 | Vietnam | NgheAn | North Central Coastal | Cattle | 27-Dec-10 | outbreak | KT153133 |
| O/VIT/2800NCVDF1202/2012 | Vietnam | QuangNinh | North Eastern | Pig | 14-Mar-12 | outbreak | KT153134 |
| O/VIT/2800NCVDF1211/2012 | Vietnam | ThaiBinh | Red River Delta | Pig | 14-Mar-12 | outbreak | KT153135 |
| O/VIT/283/2010 | Vietnam | LangSon | North Eastern | Buffalo | 31-Dec-10 | outbreak | KT153136 |
| O/VIT/30/2012 | Vietnam | SonLa | North Western | Cattle | 11-Apr-12 | outbreak | KT153137 |
| O/VIT/3155B/2012 | Vietnam | QuangNgai | South Central Coastal | Pig | 24-Mar-12 | outbreak | KT153138 |
| O/VIT/32/2012 | Vietnam | SonLa | North Western | Cattle | 11-Apr-12 | outbreak | KT153139 |
| O/VIT/33/2011 | Vietnam | LangSon | North Eastern | Buffalo | 19-Jan-11 | outbreak | KT153140 |
| O/VIT/35/2011 | Vietnam | HaNoi | Red River Delta | Pig | 21-Jan-11 | outbreak | KT153141 |
| O/VIT/352/2012pro | Vietnam | LongAn | Mekong River Delta | Cattle | 13-Apr-12 | carrier | KT153142 |
| O/VIT/366/2012pro | Vietnam | LongAn | Mekong River Delta | Cattle | 7-Jun-12 | carrier | KT153144 |
| O/VIT/381/2012pro | Vietnam | LongAn | Mekong River Delta | Cattle | 3-Jul-12 | carrier | KT153145 |
| O/VIT/3910/2012 | Vietnam | DongNai | South Eastern Vietnam | Pig | 16-Apr-12 | outbreak | KT153148 |
| O/VIT/413/2012pro | Vietnam | LongAn | Mekong River Delta | Cattle | 5-Oct-12 | carrier | KT153152 |
| O/VIT/429/2013pro | Vietnam | LongAn | Mekong River Delta | Cattle | 4-Feb-13 | carrier | KT153155 |
| O/VIT/47/2012pro | Vietnam | SonLa | North Western | Buffalo | 10-Nov-12 | carrier | KT153159 |
| O/VIT/51/2011 | Vietnam | LangSon | North Eastern | Buffalo | 26-Jan-11 | outbreak | KT153162 |
| O/VIT/52/2011 | Vietnam | LangSon | North Eastern | Buffalo | 26-Jan-11 | outbreak | KT153163 |
| O/VIT/55/2011 | Vietnam | YenBai | North Eastern | Pig | 30-Jan-11 | outbreak | KT153164 |
| O/VIT/56/2011 | Vietnam | BacKan | North Eastern | Buffalo | 8-Feb-11 | outbreak | KT153165 |
| O/VIT/63/2011 | Vietnam | TuyenQuang | North Eastern | Pig | 9-Feb-11 | outbreak | KT153170 |
| O/VIT/64124/2013 | Vietnam | TienGiang | Mekong River Delta | Pig | 17-May-13 | outbreak | KT153171 |
| O/VIT/6449/2011 | Vietnam | LongAn | Mekong River Delta | Pig | 18-Jul-11 | outbreak | KT153172 |
| O/VIT/6803/2013 | Vietnam | LongAn | Mekong River Delta | Cattle | 31-May-13 | outbreak | KT153173 |
| O/VIT/71/2011 | Vietnam | BacNinh | Red River Delta | Pig | 11-Feb-11 | outbreak | KT153174 |
| O/VIT/72/2011 | Vietnam | DienBien | North Western | Buffalo | 14-Feb-11 | outbreak | KT153175 |
| O/VIT/7223/2010 | Vietnam | LongAn | Mekong River Delta | Cattle | 1-Oct-10 | outbreak | KT153176 |
| O/VIT/77/2011 | Vietnam | ThaiNguyen | North Eastern | Pig | 15-Feb-11 | outbreak | KT153177 |
| O/VIT/85/2011 | Vietnam | TuyenQuang | North Eastern | Pig | 15-Feb-11 | outbreak | KT153178 |
| O/VIT/89/2011 | Vietnam | LaoCai | North Eastern | Buffalo | 16-Feb-11 | outbreak | KT153179 |
| O/VIT/949147/2012 | Vietnam | HoChiMinhCity | South Eastern Vietnam | Pig | 16-Jan-12 | outbreak | KT153180 |
| O/VIT/949177/2012 | Vietnam | HoChiMinhCity | South Eastern Vietnam | Pig | 16-Jan-12 | outbreak | KT153181 |
| O/VIT/98/2011 | Vietnam | HaGiang | North Eastern | Buffalo | 17-Feb-11 | outbreak | KT153182 |
| O/VIT/39/2013 | Vietnam | BacKan | North Eastern | Cattle | 24-Aug-13 | outbreak | KX944728 |
| O/VIT/24/2013 | Vietnam | BacNinh | Red River Delta | Pig | 23-Feb-13 | outbreak | KX944721 |
| O/VIT/27/2013 | Vietnam | BacNinh | Red River Delta | Pig | 3-Mar-13 | outbreak | KY492068 |
| O/VIT/37/2013 | Vietnam | BacNinh | Red River Delta | Cattle | 13-Aug-13 | outbreak | KX944727 |
| O/VIT/38/2013 | Vietnam | Daklak | Central Highlands | Cattle | 17-Aug-13 | outbreak | KY492071 |
| O/VIT/51/2013 | Vietnam | LamDong | Central Highlands | Cattle | 30-Oct-13 | outbreak | KY492073 |
| O/VIT/36/2013 | Vietnam | LangSon | North Eastern | Cattle | 1-Aug-13 | outbreak | KX944726 |
| O/VIT/24/2014 | Vietnam | LongAn | Mekong River Delta | Pig | 10-Jul-14 | outbreak | KY492075 |
| O/VIT/23/2014 | Vietnam | LongAn | Mekong River Delta | Pig | 10-Jul-14 | outbreak | *pending* |
| O/VIT/15/2013 | Vietnam | NinhThuan | South Central Coastal | Cattle | 31-May-13 | outbreak | KX944735 |
| O/VIT/16/2013 | Vietnam | NinhThuan | South Central Coastal | Cattle | 31-May-13 | outbreak | KX944717 |
| O/VIT/20/2013 | Vietnam | PhuYen | South Central Coastal | Cattle | 8-Jun-13 | outbreak | KX944718 |
| O/VIT/18/2013 | Vietnam | PhuYen | South Central Coastal | Cattle | 20-Jun-13 | outbreak | KX944720 |
| O/VIT/13/2013 | Vietnam | QuangNam | South Central Coastal | Cattle | 21-May-13 | outbreak | KX944719 |
| O/VIT/12/2013 | Vietnam | QuangNam | South Central Coastal | Cattle | 21-May-13 | outbreak | KX944715 |
| O/VIT/11/2013 | Vietnam | QuangNam | South Central Coastal | Cattle | 21-May-13 | outbreak | KY492065 |
| O/VIT/14/2013 | Vietnam | QuangNam | South Central Coastal | Cattle | 21-May-13 | outbreak | KX944714 |
| O/VIT/40/2013 | Vietnam | QuangNam | South Central Coastal | Cattle | 17-Sep-13 | outbreak | KX944716 |
| O/VIT/46/2013 | Vietnam | QuangNam | South Central Coastal | Cattle | 25-Oct-13 | outbreak | KX944729 |
| O/VIT/47/2013 | Vietnam | QuangNam | South Central Coastal | Cattle | 25-Oct-13 | outbreak | KX944731 |
| O/VIT/45/2013 | Vietnam | QuangTri | North Central Coastal | Buffalo | 23-Oct-13 | outbreak | KY492072 |
| O/VIT/22/2013 | Vietnam | SonLa | North Western | Pig | 4-Jan-13 | outbreak | KX944730 |
| O/VIT/23/2013 | Vietnam | SonLa | North Western | Pig | 4-Feb-13 | outbreak | KY492066 |
| O/VIT/25/2013 | Vietnam | SonLa | North Western | Pig | 1-Mar-13 | outbreak | KY492067 |
| O/VIT/28/2013 | Vietnam | SonLa | North Western | Pig | 8-Apr-13 | outbreak | KX944722 |
| O/VIT/30/2013 | Vietnam | SonLa | North Western | Pig | 23-May-13 | outbreak | KX944723 |
| O/VIT/31/2013 | Vietnam | SonLa | North Western | Pig | 25-May-13 | outbreak | KX944724 |
| O/VIT/32/2013 | Vietnam | SonLa | North Western | Cattle | 30-May-13 | outbreak | KY492069 |
| O/VIT/33/2013 | Vietnam | SonLa | North Western | Cattle | 30-Jun-13 | outbreak | KX944725 |
| O/VIT/11/2014 | Vietnam | SonLa | North Western | Cattle | 24-Apr-14 | outbreak | KY492070 |
| O/VIT/12/2014 | Vietnam | SonLa | North Western | Cattle | 24-Apr-14 | outbreak | KX944733 |
| O/VIT/9/2014 | Vietnam | SonLa | North Western | Cattle | 24-Apr-14 | outbreak | KY492074 |
| O/VIT/16/2014 | Vietnam | TienGiang | Mekong River Delta | Pig | 30-Apr-14 | outbreak | KX944732 |
| O/VIT/28/2014 | Vietnam | TienGiang | Mekong River Delta | Pig | 24-Jul-14 | outbreak | KX944734 |
| *Based on OIE reports, these sequences corresponded to cattle samples | | | |  |  |  |  |
| ** Published sequences without information of the clinical stage were assumed to be outbreak (clinical) samples. | | | | | |  |  |
